# Lymphangiomatosis of the ileum with perforation: A case report and review of the literature

**DOI:** 10.1016/j.amsu.2019.03.010

**Published:** 2019-03-29

**Authors:** Antonio Giuliani, Lucia Romano, Gino Coletti, Mohammad Walid A Fatayer, Giuseppe Calvisi, Francesco Maffione, Chiara Muolo, Vincenzo Vicentini, Mario Schietroma, Francesco Carlei

**Affiliations:** a Department of Surgery, University of L'Aquila, Italy; b UOC Anatomia Patologica, ASL1 Abruzzo, Ospedale San Salvatore, L'aquila, Italy

**Keywords:** Lymphangiomatosis, Bowel perforation, Small intestine, Bowel resection, Acute abdomen

## Abstract

Lymphangiomatosis is a benign proliferation of lymph vessels. Lymphatic diseases can vary from small lymphangioma to generalized lymphangiomatosis, which is a rare condition and can have several clinical manifestations. The gastrointestinal tract may be affected, but the incidence in the intestinal wall is very low. We propose in our study a case of ileal lymphangiomatosis presenting with perforation, in which the diagnosis was made after the pathological analysis of the resected intestinal tract. Although rare and not described in the literature, intestinal lymphangiomatosis could manifest itself with acute abdomen and could be a surgical urgency. This disease should be considered when intestinal perforation is observed.

## Highlights

• Lymphangiomatosis is a multisystemic disorder.
• The GI tract may be affected, but the incidence of lymphangioma of the intestinal wall is very low.
• Lymphangiomatosis of the small bowel is a rare disease, so it is easy for a clinician to make a misdiagnosis.
• Intestinal lymphangiomatosis could manifest itself with an acute abdomen and surgical urgency.
• The pathologist should keep it in mind in the differential diagnosis, when he analyses a case of perforation.

## 1 Introduction

Lymphangiomatosis is a benign proliferation of lymph vessels. Lymphatic diseases can vary from small lymphangioma to generalized lymphangiomatosis, which is a rare condition and can have several clinical manifestations [[1], [2], [3], [4], [5], [6]]. Although the underlying pathogenetic mechanism is unknown, it is generally considered as congenital malformation of the lymphatic system associated with alterations in the circulatory dynamics of the lymph [7]. It can occur anywhere in the body and also abdominal lymphangiomatosis is reported, but in many cases it involves the mesentery, omentum, mesocolon and retroperitoneum. The gastrointestinal (GI) tract may be affected, but the incidence in the intestinal wall is very low [8,9]. In the few cases described in the literature, the symptomatology was characterized mainly by abdominal pain and bleeding. Aggressive surgery should be avoided in symptomless cases, because it is now known that these lesions are benign [7].

We propose in our study a case of ileal lymphangiomatosis presenting with perforation, in which the diagnosis was made after the pathological analysis of the resected intestinal tract. In addition, the relevant medical literature on intestinal lymphangiomatosis was reviewed. This work has been reported in line with the SCARE criteria [8].

## 2 Presentation of case

A 41-year-old male presented to the Emergency Department with significant diffuse abdominal pain, nausea, vomiting and inability to pass gas or stool (constipation); the patient reported that these symptoms were present for 10 hours. He denied any significant family history of disease and he did not refer to major diseases or prior surgical interventions in his own medical history. During the physical examination, the subject was in discrete general conditions, collaborative, the sensory was intact, the decubitus indifferent, the breath eupneic and the pulse rhythmic. The vital parameters were preserved, and temperature was within normal limits. The abdominal examination revealed a flat abdomen, tender to palpation, painful to deep palpation on all quadrants, liver size appears within normal limits, Murphy's sign was negative, Blumberg's sign was positive, bowel sounds were absent. Rectal exploration indicated nothing significant. Laboratory values upon admission reported hemoglobin value of 15.5 g/dL, White Blood Cells 12.000/mmc, PCR 0.04 mg/dl. Upright abdominal films, then confirmed with computed tomography (CT) enhancement scan, revealed bowel distension and the presence of multiple gas-fluid levels. This framework suggested the presence of small bowel obstruction. As the etiology of the obstruction remained unidentified, the decision was made to perform a diagnostic laparoscopy. On entering the peritoneal cavity, the small bowel was examined: the intestinal loops were vital and vascularized. At approximately 80 cm from the ileocaecal valve, a volvulus was identified, and in close proximity there was a long tubular structure, which proved to be a Meckel's diverticulum, with adhesions to parietal peritoneum. A lysis of adhesions was performed, the Meckel's diverticulum was divided at the base using a linear stapler (45 mm) and a surgical drain was placed. On the first postoperative day, the patient showed an acute abdomen: untreatable-tensely distended abdomen, painful to deep palpation on all quadrant; Murphy's sign was negative, Blumberg's sign was positive; bowel sounds were absent. Surgical drain took out enteric material and blood. The body temperature was 38 °C. Laboratory values reported hemoglobin value of 12.7 g/dL, White Blood Cells 7.000/mmc, PCR 10.22 mg/dl. The patient again underwent urgent surgery. The intervention was an exploratory laparoscopy, then converted to open ileal resection for a microperforation of the bowel. The perforation site seemed to be distant above the previous diverticulum. About 20 cm of ileal resection was performed and side-to-side mechanical anastomosis was made. Two surgical drains were also placed. The postoperative course was regular, and the patient was discharged one the 10th post-operative day.

**Fig. 1:**
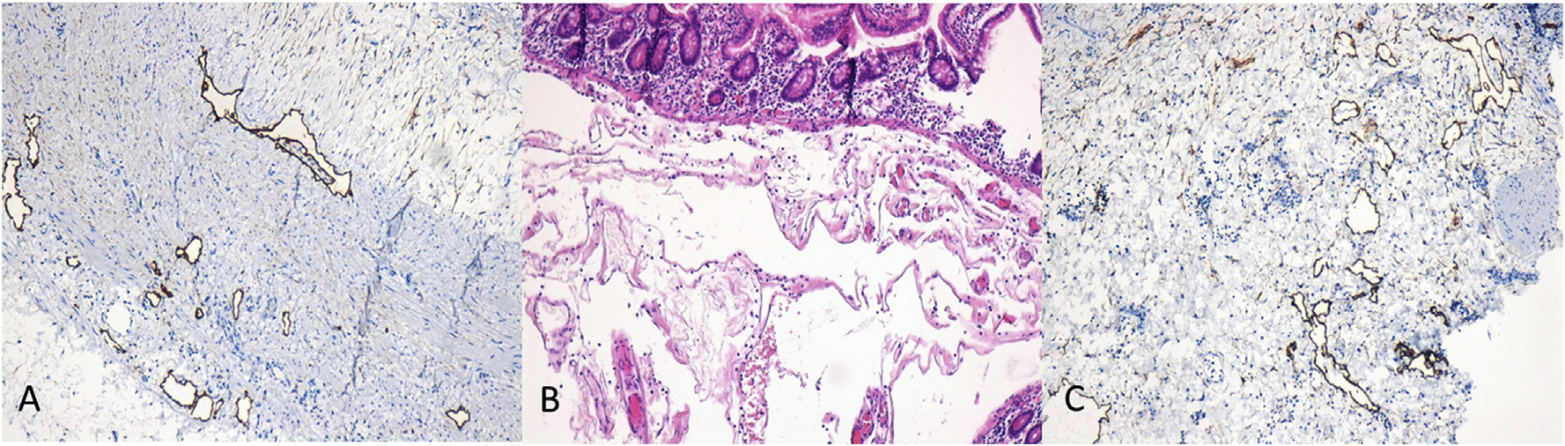
Microscopic findings. (A) Hematoxylin and eosin staining of numerous dilated lymphatic vessels (4× magnification). (B) Immunohistochemical D2-40 expression (brown color) in dilated lymphatic vessels of the submucosa (10× magnification). (C) Subserous dilated lymphatic vessels with discontinuity of the muscular layer and serositis (H&E, 10× magnification). (For interpretation of the references to color in this figure legend, the reader is referred to the Web version of this article.)

**Fig. 2:**
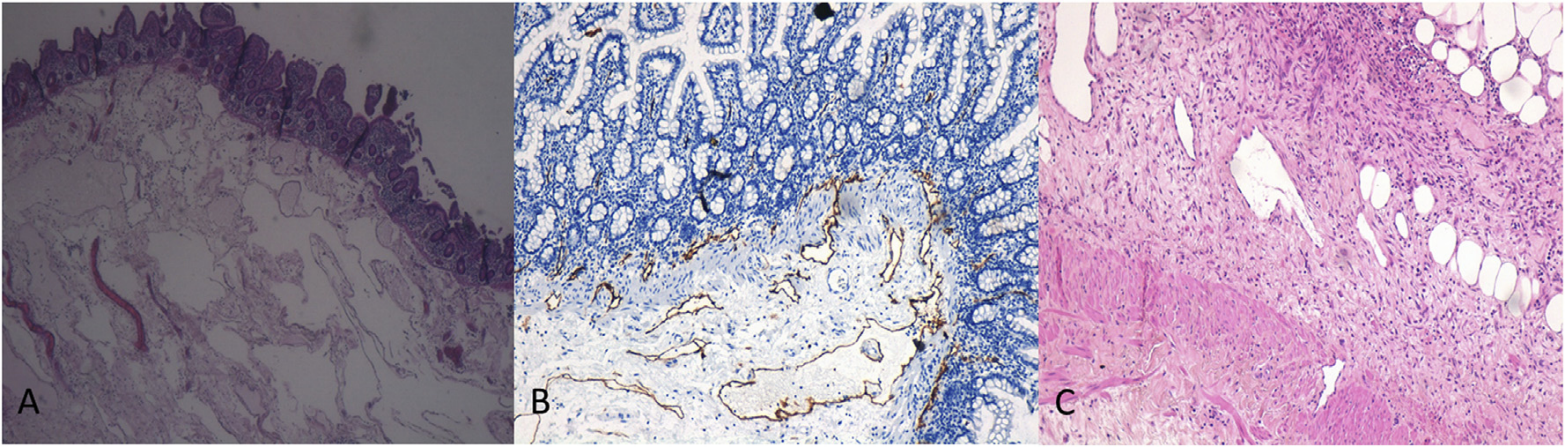
(A) D2-40 immunostaining shows positive reactivity for lining endothelial cells of lymphatic spaces in the muscular layer (10× magnification). (B) Numerous submucosal dilated lymphatic vessels (H&E, 10× magnification). (C) Lymphatic vessels that interrupt muscular layer (D2-40 stain, 10× magnification).

Histological examination showed stratification of fibrin and granulocytes on the serosa and presence of diffuse lymphangiomatosis (positive immunohistochemistry of D2-40 marker) involving the submucosa and, in some parts, the full-thickness muscular wall (Fig. 1, Fig. 2).

## 3 Discussion

Lymphangiomatosis is a multisystemic disorder, characterized by congenital malformation of the lymphatic system with channels and cystic spaces of varying size. It may affect all the areas of the body; therefore, symptoms and complications are related to localization. Usually in the course of the disease patients are asymptomatic at first, but then the abnormally proliferating lymphatic channels are capable of massive expansion and infiltration into surrounding tissues. Abdominal lymphangiomatosis is quite often reported, but in many cases it arises in the mesentery, omentum, mesocolon and retroperitoneum. The GI tract may be affected, but the incidence of lymphangioma of the intestinal wall is very low, and it is even rarer in the small bowel (less than 1%) [9,10]. To the best of our knowledge, all cases reported in the literature with GI tract involvement are summarized in Table 1 [1,2,7,[10], [11], [12], [13], [14], [15], [16], [17], [18]].

**Table 1:** Papers about lymphangiomatosis-related disease.

Reference	Age and gender	GI tract organs involved	Type of lesions	Histopathologic findings	Clinical features	Diagnostic workup	Treatment	Follow-up and outcome
Valakada J, Madhusudhan KS et al. [1]	59-years-old woman	duodenum, jejunum, mesentery and retroperitoneum	marked thickening of the small bowel loops in the duodenum and jejunum and multiple tubular channels in the mesentery and retroperitoneum hyperintense on T2-weighted images	lymphangectasia	recurrent abdominal pain, multiple episodes of melena, pedal edema, pallor and mild hepatosplenomegaly	abdominal magnetic resonance imaging (MRI), abdominal ultrasonography, double-balloon enteroscopy and biopsy	conservative management: low-fat and high-protein diet	
Lin RJ, Zou H et al. [2]	38- year-old female	fundus of the stomach, peripancreatic area, mesenteric area, retroperitoneal space of the spleen, right upper quadrant of the greater omentum	multiple small cystic lesions without enhancement (TC), multiple cystic dark areas (abdominal ultrasonography)	submucosal microscopic cysts of lymphatic channels with walls composed of thin fibrous tissue	melena for 3 months, weakness for 10 days, hemoptysis for 4 months	computed tomography, abdominal ultrasonography, biopsy	distal gastric resection and Billroth II-type anastomosis	she continued to present melena, iron deficiency anemia and hypoproteinemia after the surgery
Jung SW, Cha JM et al. [7]	31-years-old woman	ascending colon, from the cecum to the hepatic flexure	multiple thumbprint-like lesions on the air contrast barium enema; clusters of round submucosal tumors with smooth surface, without ulcerations or erosions and positive to the cushion sign on the colonscopy; the EUS showed echo free cysts with a clear border and septal walls in the sbmucosal layer	submucosal cysts lined by endothelial cells, serous liquid resembling lymphatic fluid, with occasional multinucleated cells and without fat or blood cell components		air contrast barium enema, colonscopy, EUS and endoscopic biopsy	the patient was not treated with invasive treatment because she was asymptomatic	
Rai P, Rao RN et al. [10]	31-years-old man	small bowel and small bowel mesentery starting from mid-jejunum to ileocecal junction	protruding submucosal lesions on the colonscopy, cystic lesions on the CT	multiple irregular dilated space lined by endothelial cells with lymphoid aggregates, filled with acellular proteinacious material and no evidence of malignant cells	recurrent melena for the last 8 years and iron deficiency	colonscopy, capsule andoscopy, contrast-enhanced CT, laparatomy with intraoperative endoscopy and endoscopic biopsy	limited ileocecal resection, end ileostomy and distal mucus fistula. After few days continuity was restored.	no gastrointestinal bleed, haemoglobin and albumin were normalised
Hwang SS, Choi HJ et al. [11]	71-year-old man	jejunal and adjacent mesentery	multiple nodular mesenteric masses infiltrating into the jejunum and adjacent mesentery; multiloculated cystic lesion from the mucosa to the subsierosa	numerous multiloculated, cystically dilated spaces lined by attenuated endothelium that appeared to dissect through the muscolaris propria of the small intestine with inside fluid containing lymphocytes		computed tomography, 18FDG PET/CT, biopsy	complete surgical resection of the segment involving the lesions	
Ilhan M, Oner G et al. [12]	43-years-old woman	ileum and jejunum	diffuse wall thickness (CT)	expanded cystic vascular lesions, partly extending to the intestinal mucosa and subserosa	weakness, swelling in leg, weight loss, pretibial edema and recurrent upper respiratory infections	colonscopy, abdominal ultrasound, computed tomography, PET-CT and biopsy	resection of the affected part of ileum and end-to-end anastomosis; lymph node in the mesentery of 35–45 cm to the proximal terminal ileum were excised	after 1 month surgery pretibial edema was non seen, protein and albumin increased
Chung WC, Kim HK et al. [13]	48-years-old man	proximal transverse colon	several protruding mucosal lesions covered with normal mucosa on the colonscopy	cystic lesions with a lumen covered by a single layer of flat endothelial cells	abdominal discomfort and anemia	colonscopy, abdomen CT, biopsy	endoscopic mucosectomy	the patient had abdominal pain and anemia when he was followed up 3 month after musectomy
Lee JS, Kim GW et al. [14]	38 year-old man for a general check-up	mid-portion of the ascending colon up to the proximal portion of the tansverse colon	variably sized cystic mass lesions	normal colonic mucosa and markedly dilated lymphatic vessels in the submucosa positive at immunohistochemical staining for CD34 and D2-40 (marker of vascular endothelium and lymphatic endothelium)		chest and abdominal radiography, esophagogastroduodenoscopy, colonscopy, abdominal ultrasonography, CT and biopsy	several incisions and excisional biopsies	no complications such as bleeding or protein-losing enteropathy were noticed
Fang JF, Qiu LF et al. [15]	57-years-old woman	small intestine, 30 cm distal to the flexor tendon	mass with ulcers and erosion approximately of 5 cm × 4 cm	intrinsic layer of dilated lymphatic vessels and a small amount of interstitial neutrophil, eosinophil, plasma cell infiltration	recurrent melena for more than 2 months	gastroscopy, enteroscopy, and biopsy	partial resection of the small intestine	during the follow-up no recurrence was observed
Dong A, Zhang L et al. [16]	22-years-old female	mesentery and ileum	mass involving mesentery and ileum with nodules in the mass	proliferation and dilation of the mucosal lymphatic, containing a large amount of red blood cells. The cells were positive for CD31, CD34 and D2-40. Ki-67 was about 1%.	9-month history of intermittent melena, weakness and palpitation	abdominal MR, abdominal CT, PET-CT and biopsy	resection of the abdominal mass and a segment of 60 cm of the ileum invaded by the abdominal mass	after surgery symptoms improved and follow-up laboratory tests showed normal red blood cell count and hemoglobin level
Lu G, Li H et al. [17]	79-year-old man	sigmoid colon	multiple cystic masses (colonscopy), with spetal walls in the submucosal layer	cysts located in the submucosal layer surrounded by flat endothelial cells that were positive for D2-40 at the immunoistochemistry	intermittent attacks of bowel bleeding and abdominal discomforts for 3 months	colonscopy, endoscopic ultrasound and biopsy	laparoscopy-assisted partial sigmoid colon resection	in the 2-year follow-up after the operation, no bleeding or other complications were noticed
Xue L, Guo WG et al. [18]	58-year-old man	lower esophagus	longitudinally protruding mass covered with normal esophageal mucosa and a lesion outside but adjacent to the wall of the esophagus	multiple dilated lymphatic vessels of a different sizes filled with pink beneath squamous epithelium	dysphagia of 7 months	esophagogastroscopy, esophageal ultrasonography, chest CT and biopsy	a right lateral thoracotomy was performed fot the resection of the cysts, first the lesion outside and than that protruding in to the esophageal lumen	the postoperative course was uneventful and at the patient was discharged on th 10th postoperative day

There is only one case report of lymphangiomatosis involving the oesophagus [18] and one case report involving fundus of the stomach [2]. Lymphangiomatosis of the colon is described in four papers [7,[13], [14], [15]]. In these patients, the main symptoms were abdominal discomfort, bleeding and anaemia.

Lymphangiomatosis of small bowel is described in 6 works [1,[10], [11], [12],^15^,^16^]. Patients had abdominal pain, bleeding, melena, anaemia, pedal edema, weakness and weight loss, but no case of perforation is reported.

In the majority of patients described in the literature, diagnosis was suggested by colonoscopy and biopsy, which mainly showed protruding submucosal lesions. The diagnostic workup was sometimes integrated by contrast-enhanced CT, showing marked thickening of the walls of the bowel loops.

In five papers [[10], [11], [12],^15^,^16^], patients were treated with surgical resection of the affected part of intestine, whereas one patient improved on conservative management and was put on low-fat and high-protein diet [1]. In all reported cases, follow-up was of short duration. In two cases patients continued to have symptoms after surgery [2] or after endoscopic mucosectomy [13].

The histological features of the lymphangiomatosis are non-specific, and the definitive diagnosis requires the demonstration of an hyperproliferation of normal lymphatic vessels with normal endothelium, predominantly in the context of submucosa, with disruption of the muscular layer and sometimes of the serosa. This condition creates a *locus minoris resistentiae,* and this may explain the pathogenesis of the perforation, reported in our case. The involvement can be continuous or, more frequently, segmental. The impairment of the muscular layer and of the submucosal nerve plexus could also have contributed to the development of the intestinal obstruction, which was the symptom because our patient came to our attention. This suggests that also lymphangiomatosis should be taken into account among other rare causes of intestinal obstruction [19,20].

## 4 Conclusion

In conclusion, lymphangiomatosis of the small bowel is a rare disease that has no specific clinical features, so it is easy for a clinician to make a misdiagnosis or to miss diagnosis. In some cases, surgical resection may be required to provide definitive histological diagnosis, as occurred in our cases. We want to share our experience about this, because, although rare and not described in the literature, intestinal lymphangiomatosis could manifest itself with an acute abdomen and surgical urgency. This disease should be considered when intestinal perforation is observed. In particular, the pathologist should keep it in mind in the differential diagnosis, when he analyses a case of perforation whose cause is not very clear and specified.

## Ethical approval

Ethical approval was not required.

## Sources of funding

No funding

## Author contribution

Antonio Giuliani, Lucia Romano: Writing.

Gino Coletti, Mohammad Walid A Fatayer, Giuseppe Calvisi: Images and contribution to the text.

Francesco Maffione, Chiara Muolo, Vincenzo Vicentini: Data collection.

Mario Schietroma, Francesco Carlei: Study design and review.

## Conflicts of interest

No conflict of interest.

## Research registration number

None.

## Guarantor

Prof. Francesco Carlei.

## Consent

Written informed consent was obtained from the patient for publication of this case report and accompanying images. A copy of the written consent is available for review by the Editor-in-Chief of this journal on request.

## Provenance and peer review

Not commissioned, externally peer reviewed.

## References

[1] Valakada J., Madhusudhan K.S., Ranjan g (2018 May - Jun). Abdominal lymphangiomatosis with intestinal lymphangiectasia diagnosed by magnetic resonance lymphangiography: a case report. Curr. Probl. Diagn. Radiol..

[2] Lin R.-Y., Zou H., Chen T.-Z. (2014). Abdominal lymphangiomatosis in a 38-year-old female: case report and literature review. World J. Gastroenterol..

[3] Won K.C., Jang B.I., Kim T.N. (1993). A case of primary intestinal lymphangiectasia. Korean J. Intern. Med..

[4] Yang D.H., Goo H.W. (2006). Generalized lymphangiomatosis: radiologic findings in three pediatric patients. Korean J. Radiol..

[5] Puri A.S., Aggarwal R., Gupta R.K. (1992). Intestinal lymphangiectasia: evaluation by CT and scintigraphy. Gastrointest. Radiol..

[6] Blei F. (2011). Lymphangiomatosis: clinical overview. Lymphatic Res. Biol..

[7] Jung S.W., Cha J.M., Lee J.I. (2010 Jan). A case report with lymphangiomatosis of the colon. J. Korean Med. Sci..

[8] Agha Riaz A., Borrelli Mimi R., Farwana Reem, Koshy Kiron, Fowler Alexander J., Orgill Dennis P. (2018). For the SCARE Group. The SCARE 2018 statement: updating consensus Surgical CAse REport (SCARE) guidelines. Int. J. Surg..

[9] Matsuda T., Matsutani T., Tsuchiya Y. (2001). A clinical evaluation of lymphangioma of the large intestine: a case presentation of lymphangioma of the descending colon and a review of 279 Japanese cases. J. Nippon Med. Sch..

[10] Rai P., Rao R.N., Chakraborthy S.B. (2013 Apr 19). Caecal lymphangioma: a rare cause of gastrointestinal blood loss. BMJ Case Rep..

[11] Hwang S.S., Choi H.J., Park S.Y. (2009 Aug 21). Cavernous mesenteric lymphangiomatosis mimicking metastasis in a patient with rectal cancer: a case report. World J. Gastroenterol..

[12] Ilhan M., Oner G., Alibeyoglu A. (2016 Aug 17). Primary intestinal lymphangiomatosis of the ileum in an adult-the role of surgical approach. J. Surg. Case Rep..

[13] Chung W.C., Kim H.K., Yoo J.Y. (2008 Oct 7). Colonic lymphangiomatosis associated with anemia. World J. Gastroenterol..

[14] Lee Y.S., Kim G.W., Cho H.J. (2015 Jan). Colonic lymphangiomatosis resolved after excisional biopsy. Clin. Endosc..

[15] Fang Y.F., Qiu L.F., Du Y. (2012 May 7). Small intestinal hemolymphangioma with bleeding: a case report. World J. Gastroenterol..

[16] Dong A., Zhang L., Wang Y. (2016 Feb). Abdominal kaposiform hemangioendothelioma associated with lymphangiomatosis involving mesentery and ileum: a case report of MRI, CT, and 18F-FDG PET/CT findings. Medicine (Baltim.).

[17] Lu G., Li H., Li Y. (2017 Jan). Lymphangiomatosis of the sigmoid colon - a rare cause of lower gastrointestinal bleeding: a case report and review of the literature. Oncol. Lett..

[18] Xue L., Guo W.G., Hou J. (2012 Jun). Huge lymphangiomatosis of the esophagus. Ann. Thorac. Surg..

[19] De Santis G., Sista F., Giuliani A. (2011 Sep-Oct). Idiopathic intramural hematoma of sigmoid colon. A case report. Ann. Ital. Chir..

[20] de Vries J.J., Vogten J.M., de Bruin P.C. (2007). Mesenterical lymphangiomatosis causing volvulus and intestinal obstruction. Lymphatic Res. Biol..

